# Harnessing Qatar Biobank to understand type 2 diabetes and obesity in adult Qataris from the First Qatar Biobank Project

**DOI:** 10.1186/s12967-018-1472-0

**Published:** 2018-04-12

**Authors:** Ehsan Ullah, Raghvendra Mall, Reda Rawi, Naima Moustaid-Moussa, Adeel A. Butt, Halima Bensmail

**Affiliations:** 1 Qatar Computing Research Institute, Hamad Bin Khalifa University, Doha, Qatar; 2 Vaccine Research Center, National Institute of Allergy and Infectious Diseases, National Institutes of Health, Bethesda, MD 20814 USA; 3 Obesity Research Cluster (ORC), Nutrigenomics, Inflammation and Obesity Research (NIOR) Laboratory, Texas Tech University, 1301 Akron Street, Lubbock, TX 79409-1270 USA; 4 Department of Medicine, Weill Cornell Medical College, Doha, Qatar; 5 Department of Healthcare Policy and Research, Weil Cornell Medical College, Doha, Qatar; 6 Department of Medicine, Clinical Epidemiology Research Unit Hamad Medical Corporation, Doha, Qatar

**Keywords:** Qatar Biobank, Diabetes, Obesity, Biostatistics, Epidemiology, Machine learning

## Abstract

**Background:**

Human tissues are invaluable resources for researchers worldwide. Biobanks are repositories of such human tissues and can have a strategic importance for genetic research, clinical care, and future discoveries and treatments. One of the aims of Qatar Biobank is to improve the understanding and treatment of common diseases afflicting Qatari population such as obesity and diabetes.

**Methods:**

In this study we apply a panorama of state-of-the-art statistical methods and machine learning algorithms to investigate associations and risk factors for diabetes and obesity on a sample of 1000 Qatari population.

**Results:**

Regarding diabetes, we identified pronounced associations and risk factors in Qatari population including magnesium, chloride, c-peptide of insulin, insulin, and uric acid. Similarly, for obesity, significant associations and risk factors include insulin, c-peptide of insulin, albumin, and uric acid. Moreover, our study has revealed interactions of hypomagnesemia with HDL-C, triglycerides, and free thyroxine.

**Conclusions:**

Our study strongly confirms known associations and risk factors associated with diabetes and obesity in Qatari population as previously found in other population studies in different parts of the world. Moreover, interactions of hypomagnesemia with other associations and risk factors merit further investigations.

**Electronic supplementary material:**

The online version of this article (10.1186/s12967-018-1472-0) contains supplementary material, which is available to authorized users.

## Background

Chronic diseases such as diabetes, obesity and cancer are caused by the complex interaction between environmental factors (such as diet, lifestyle, and the built environment) and genetic factors [1–3]. To understand the ultimate role of environmental, behavioral, and genetic factors along with their interactions, large-scale population cohorts have been established, mainly in Europe, North America, China, Japan, and Korea [4]. No such large population-based studies currently exist in the Gulf Region [5].

Two large biobank projects were launched, one in Saudi Arabia by the King Abdullah International Medical Research Center’s (KAIMRC) and the second in Qatar, by the Qatar Foundation and the Supreme Council of Health. The Qatar Biobank is a Qatar national population based prospective cohort study which includes the collection of biological samples, with long-term storage of data and samples for future research. The ultimate goal is to allow physicians and researchers to use the data collected from the biobank to conduct a large-scale study of the combined effects of genes, environment, and lifestyle on these diseases, to educate people on risk factors for these common diseases and to study disease incidence patterns and develop new diagnostic and therapeutic approaches. Using this pilot data, we had access to 60 features measured on 1000 Qatari citizens. The variables summarize physical, clinical and biochemical measurements such as age, gender, ethnicity, albumin, transaminase time, calcium, cholesterol, and uric acid.

The aim of this study is to use state-of-the-art statistical and machine learning methods to identify biomarkers for medical conditions; diabetes and obesity in this case, to identify the associated risk factors in Qatari population compared to those previously found in other studies. To the best of our knowledge, this is the first study that has been done on Qatari biobank few months after its release.

## Methods

### Ethical approval

The study was conducted according to the policies, regulations and guidelines for Research Involving Human of the Qatar Ministry of Public Health. All procedures involving human subjects were approved by the Institutional Review Board of Hamad Medical Corporation in Doha, Qatar. Written informed consent was obtained from all participants prior to their enrollment in the study.

### Study population

The Qatar Biobank project is a population based cohort, aiming to prospectively examine 60,000 Qataris and long term residents (≥ 15 years living in Qatar) aged 18 years or more. Details are available in [6]. Briefly, potential participants were contacted via word of mouth or via Qatar Biobank’s website www.qatarbiobank.org.qa. Consented participants visited Qatar Biobank facility at Hamad Medical City Building 17, Doha, Qatar, where they underwent a 5-stage interview, physical and clinic measurement sequence, with an average duration of 3 h. Extensive questionnaires (i.e. health behaviors, medical history, lifestyle characteristics, physical activity, mental health, environmental exposures etc.) and clinical examination (i.e. anthropometric measurements, blood pressure, electrocardiogram, bone density etc.) were administered by trained research personnel at enrollment. Participants were asked to provide biological samples (blood, urine and saliva). Biological samples were sent for analysis at the diagnostic laboratories at Hamad Medical Corporation, Doha, Qatar. All lab equipment was calibrated to ensure precision of results. The measured features comprise of routinely measured clinical biomarkers, for details see [6]. Qatar Biobank is recruiting more participants after completion of the pilot study to be as representative as possible of the eligible Qatari population, with a target of 60,000 study participants [6].

Out of the participants, data of 1305 randomly selected participants was used for the present pilot project. The participants consisted of 661 males (50.65%) and 644 females (49.35%), of which 99% were Qataris and remaining 1% were non-Qatari long term residents. The variables having more than 50% missing values and subjects having more than 9 missing values were removed. The dataset was used for two studies: diabetes and obesity. We denote the samples as dataset ${\mathbf{D}_{\mathbf{t2d}}}$ for diabetes analysis. The samples were divided into two groups: cases (*n* = 312 subjects having HbA1C% $\ge$6.5) and controls (*n* = 898 subjects having HbA1C% < 6.5). For obesity analysis, the dataset ${\mathbf{D}_{\mathbf{obs}}}$ was divided into two groups: cases (*n* = 508 subjects with BMI ≥ 25 kg/m^2^) and controls (*n* = 224 subjects with 18 ≤ BMI < 25 kg/m^2^).

### Missing value imputation

We identified that 2.81% values of the diabetes dataset and 2.64% values of the obesity dataset were missing. Instead of removing the missing values we decided to approximate missing values using the well-known technique multivariate imputation by chained equations (MICE) implemented in the R package *mice* [7].

### Baseline statistics

The baseline statistics for the two groups of samples were computed using R [8]. First, normality of the variables was tested using Anderson–Darling test in *nortest* package of R [9]. For a normally distributed variable in both groups, Student’s t-test was used to determine significance of difference in the group means. In this case, the group variance of the variable was calculated using F test. For remaining variables, Mann–Whitney test was used to determine significance of difference in the group means. A reported P value lower than 0.05 indicates the corresponding variable is statistically different in the groups.

### Regularization models

In this paper, we have used the elastic net, the glinternet, the lasso projection and hdi methods for linear regression models.

#### The elastic net

The elastic net is a lasso based statistical method that combines L^2^ penalty with L^1^ penalty [10]. The elastic net is a better method compared to lasso as the lasso selects only one variable (randomly) out of a group of variables having high pairwise correlation. We used R package *glmnet* [11] for computation of coefficients with 10-fold cross validation for training the elastic net model.

One of the drawbacks of the elastic net is that it does not calculate statistical significance of the variables (P values), which motivated us to use methods other than the elastic net as well.

#### Glinternet

The glinternet is a group-lasso based method developed by Lim and Hastie [12]. The method learns pairwise interactions of variables in linear regression models satisfying strong hierarchy. An interesting feature of this method is its ability to incorporate both continuous and categorical variables at the same time in the model making it a unique method to analyze mixed data. We used R package *glinternet* [13] for computation of coefficients with tenfold cross validation for training the glinternet interaction model.

#### The lasso projection

The lasso projection (lasso proj) or de-sparsified lasso is a regularization based method that performs statistical inference of low dimensional parameters with high dimensional data [14]. The method uses low dimension projection approach to construct confidence intervals for the estimated regression parameters. Bühlmann and van de Geer improved the de-sparsified lasso by incorporating misspecifications in linear regression models [15]. We used R package *hdi* [16] for P value calculations for the lasso projection method.

#### High-dimensional inference

In case of high-dimensional data $p>n$, standard covariance tests cannot be used without an estimate of the error standard deviation ($\Sigma ^2$). Meinshausen et al. introduced a method for computation of P values and confidence intervals in high-dimensional data [17]. In their approach, the data is split into two groups. Variables are selected in one group using the lasso regularization (the elastic net with tenfold cross validation). The selected variables are then used as predictors in an ordinary least squared regression on the other group to obtain associated P values. We used R package *hdi* [16] for P value calculation.

### Machine learning models

In this section, we briefly summarize the modelling techniques used to generate predictive models and unsupervised clustering methods for the datasets ${\mathbf{D}_{\mathbf{t2d}}}$ and ${\mathbf{D}_{\mathbf{obs}}}$. Our goal is to identify variables, which helps to differentiate cases from controls in the two datasets. For this purpose we used two predictive modelling techniques namely random-forests and gradient boosting machines (GBM), which can capture non-linear interactions and produce models which are interpretable. These models not only provide the importance of each variable w.r.t. the phenotype but also classify unseen samples to cases and controls. We have reported the importance of variables in the predictive models computed by R package *caret* [18]. The importance of variables was ranked and scaled to a maximum importance of 100 for comparison between different methods. The details of machine learning methods is available in Additional file 1.

#### Random forests

Random forest belongs to the class of ensemble based supervised learning techniques [19]. Random forest algorithm applies the general technique of bagging or bootstrapped aggregating [20] to decision tree learners. By performing this bootstrapping procedure, we obtain better model performance as it decreases the variance of the model, without increasing bias. This means that though each tree is a weak learner and sensitive to noise within its respective data, the average/majority of many trees is not, as long as the trees are not correlated. Thus, this bootstrap sampling is used to de-correlate the trees by showing them different parts of the dataset. Random forests automatically rank the importance of variables in a classification problem by considering the average Information Gain [19] corresponding to each variable for all the trees. We used R package *caret* [18] to generate random forest models.

#### Gradient boosting machine

We used gradient boosting machine another ensemble technique for building a predictive model [21–23]. The principle idea behind this algorithm is to construct the new base-learners to be maximally correlated with the negative gradient of the loss function, associated with the whole ensemble. We used R package *caret* [18] for building a GBM predictive model. Detailed description of the method is provided in [22] and Additional file 1.

#### Unsupervised learning

We used principal component analysis to perform exploratory analysis to identify variables that contribute to the maximum variance in the data. Such variables can be used as potential biomarkers to classify a new sample as case or control. We have used pca biplots [24] to provide visualization of the variables along with the samples. We used R package *stats* for building pca biplots [24]. We performed principal component analysis (PCA) using top ten discriminative variables from machine learning methods mentioned above. The plots represent contribution of each variable in the PCs in form of labeled vectors. The angle between two vectors indicates the correlation of the variables. In these plots the colored ellipses represent the density of the two classes.

### Survival and risk analysis

#### Survival analysis

**Fig. 1:**
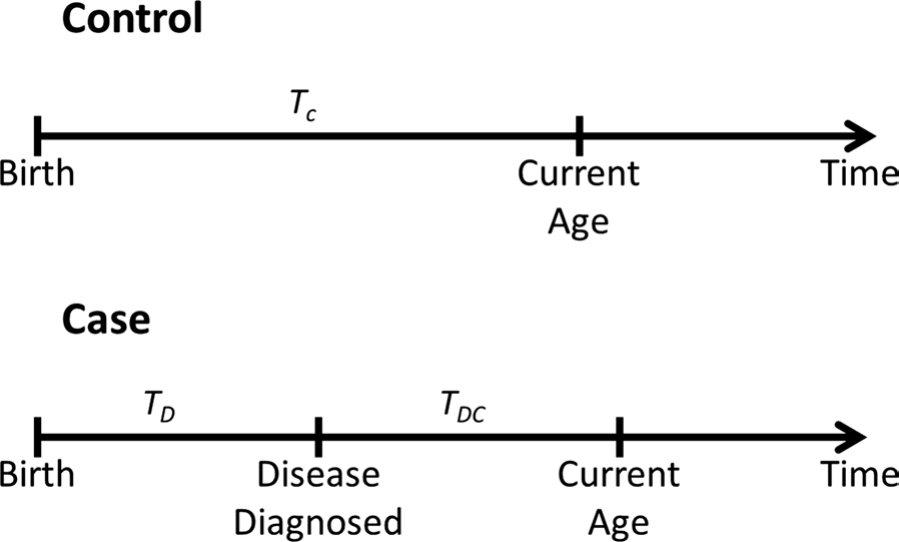
Prognosis of diabetes in controls and cases

We have applied survival analysis on the prognosis of diabetes in the Qatari population. Survival analysis [25, 26] examines and models the time it takes for events to occur, diabetes in our case. Survival analysis focuses on the distribution of event times. In our analysis, we used it to estimate the distribution of time of diabetes development. The time in the model is considered with reference to the time of birth as shown in Fig. 1. For controls, since diabetes is not developed to the current age, the time is considered to be equal to the current age T_C_ and the data is considered to be right censored as the future time of diabetes development is not known. For cases, the time is considered to be equal to the time of event T_D_, which is the diagnosis of diabetes. We have used the Kaplan–Meier estimator [27] implemented in the R package *survival* [28] to estimate the distribution of time of diabetes development.

#### Risk analysis

We have also analyzed event times using Cox proportional hazard model [29], a regression based model, in our study. The model assumes covariates to be linear in the log space. Moreover, the model assumes exponential hazard distribution [30] or constant hazard function i.e. the survival function changes proportionally with each variable. We have performed cox proportional hazard regression analysis for each of the predictor variable independent of the other and also in a multivariate regression. We have used the R package *survival* [28] for cox proportional hazard regression analysis.

## Results

We have applied the aforementioned methods on the study population considering all the participants. We have also performed gender stratified analysis to investigate the impact of gender (see Additional file 2 for details).

### Baseline characteristics of the study population

Based on the baseline statistics, age was found very significantly associated with diabetes and obesity. Therefore, age was removed from the dataset and phenotype was age adjusted for rest of the analysis. The baseline characteristics of ten most significant variables differentiating the study population for diabetes and obesity are listed in Table 1. Complete list of baseline characteristics is available in Additional file 3. Triglycerides, BMI, and vitamin D were significantly higher (P values $2.03\times 10^{-11}$, $8.00\times 10^{-09}$, and $1.93\times 10^{-08}$ respectively) whereas chloride, magnesium, albumin, free triiodothyronine, sodium and high density lipoprotein were significantly lower (P values $4.51\times 10^{-24}$, $3.50\times 10^{-23}$, $1.07\times 10^{-10}$, $1.50\times 10^{-08}$, $2.17\times 10^{-08},$ and $5.25\times 10^{-08}$ respectively) in cases compared to controls in the diabetes dataset. Similarly, c-peptide of insulin, triglycerides, HBA1C%, insulin, and uric acid were significantly higher (P-values $1.95\times 10^{-28}$, $6.94\times 10^{-25}$, $1.43\times 10^{-20}$, $5.19\times 10^{-15}$, $6.87\times 10^{-13}$, $1.54\times 10^{-10}$, and $4.25\times 10^{-08}$ respectively) whereas albumin, high density lipoprotein, magnesium, and total bilirubin were significantly lower (P values $3.24\times 10^{-10}$, $3.61\times 10^{-08}$ , and $7.18\times 10^{-08}$ respectively) in cases compared to controls in the obesity dataset.

**Table 1:** Baseline characteristics for diabetes and obesity study

	Case (n = 312)	Control (n = 898)	P value
Diabetes study			
Age (years)	50.99 ± 10.33	39.01 ± 12.13	8.60 × 10^−55^
Chloride (mmol/L)	99.44 ± 2.61	101.18 ± 1.99	8.60 × 10^−24^
Magnesium (mmol/L)	0.79 ± 0.08	0.84 ± 0.66	3.50 × 10^−23^
Triglycerides (mmol/L)	1.83 ± 0.96	1.39 ±1.00	2.03 × 10^−11^
Albumin (g/L)	44.25 ± 2.85	45.47 ± 2.86	1.07 × 10^−10^
BMI	31.39 ± 5.87	29.11 ± 6.00	8.00 × 10^−09^
Free triiodothyronine (pmol/L)	4.31 ± 0.69	4.57 ± 0.62	1.50 × 10^−08^
Vitamin D (ng/L)	21.69 ± 9.65	18.17 ± 9.40	1.93 × 10^−08^
Sodium (mmol/L)	139.38 ± 2.54	140.30 ± 2.25	2.17 × 10^−08^
High density lipoprotein (mmol/L)	1.21 ± 0.33	1.34 ± 0.36	5.25 × 10^−08^

	Case (n = 508)	Control (n = 224)	P value
Obesity study			
Albumin (g/L)	44.07 ± 2.76	46.58 ± 2.61	1.95 × 10^−28^
Age (years)	45.36 ± 11.77	35.02 ± 12.68	6.94 × 10^−25^
C-peptide of insulin (ng/L)	3.43 ± 2.07	2.17 ± 1.39	1.43 × 10^−25^
Triglycerides (mmol/L)	1.61 ± 1.10	1.10 ± 0.62	5.19 × 10^−15^
HBA1C%	6.53 ± 1.65	5.71 ± 1.26	6.87 × 10^−13^
Insulin (mcunit/mL)	22.77 ± 38.35	10.59 ± 10.95	1.54 × 10^−10^
High density lipoprotein (mmol/L)	1.27 ± 0.33	1.45 ± 0.36	3.24 × 10^−08^
Magnesium (mmol/L)	0.81 ± 0.07	0.84 ± 0.06	3.61 × 10^−08^
Uric acid (umol/L)	304.39 ± 80.52	272.01 ± 68.71	4.25 × 10^−08^
Total blirubin (umol/L)	6.19 ± 3.76	8.23 ± 4.94	7.18 × 10^−08^

Rows are sorted by significance, ten most significant variables are reported

### Regularization models

Results of the elastic net, the glinternet, the lasso proj and hdi are listed in Table 2 for diabetes and obesity studies. Coefficients ($\beta$) are reported for the elastic net and glinternet whereas P values are reported for the lasso proj and hdi. A positive coefficient indicates correlation whereas a negative coefficient indicates inverse correlation of the variable with the phenotype.

**Table 2:** Significant results of elastic net, glinternet, lasso proj and hdi

	Elastic net	Glinternet	Lasso proj	hdi
	Coefficient (*β*)	Coefficient (*β*)	P value	P value
Diabetes study				
Magnesium	− 1.01 ×10^−00^	− 2.82 ×10^−00^	3.35 × 10^−10^	2.34 × 10^−09^
Calcium	1.33 × 10^−01^	− 3.07 × 10^−02^	5.61 × 10^−02^	
High density lipoprotein	− 1.19 × 10^−01^	− 5.16 × 10^−01^	3.73 × 10^−03^	6.96 × 10^−01^
Phosphorus	6.47 × 10 ^−02^	− 8.15 × 10^−03^	4.71 × 10^−01^	
Chloride	− 3.48 ×10^−02^	− 1.66 × 10^−02^	2.99 × 10^−09^	7.43 × 10^−11^
Free triiodothyronine	− 3.05 × 10^−02^	− 1.08 × 10^−01^	2.58 × 10^−03^	
Albumin	− 1.08 × 10^−03^	1.29 × 10^−03^	2.09 × 10^−01^	
Insulin	9.95 × 10^−04^	2.93 × 10^−04^	1.88 × 10^−04^	9.36 × 10^−02^
Uric acid	− 5.40 ×10^−04^	− 3.32 × 10^−03^	1.31 × 10^−05^	4.05 × 10^−04^
Obesity study				
Magnesium	− 2.00 × 10^−01^	− 2.79 × 10^−02^	6.55 × 10^−01^	
High density lipoprotein	− 8.10 × 10^−02^	4.49 × 10^−01^	7.46 × 10^−03^	
Albumin	−3.00 × 10^−02^	− 7.36 × 10^−02^	1.11 × 10^−05^	2.40 × 10^−09^
Calcium	− 2.65 × 10^−02^	− 2.06 × 10^−01^		
C-peptide of insulin	1.74 × 10^−02^	− 5.30 × 10^−02^	1.18 × 10^−01^	3.27 × 10^−01^
Cholesterol	1.11 × 10^−02^	1.59 × 10^−02^	4.83 × 10^−01^	
Total bilirubin	− 3.30× 10^−03^	4.52 × 10^−02^		
Vitamin D	− 3.16 × 10^−03^	− 2.72 × 10^−02^	1.03 × 10^−03^	1.09 × 10^−01^
Triglycerides	2.51 × 10^−03^	− 1.01 × 10^−01^		
Uric acid	5.87 × 10^−04^	− 4.61 × 10^−03^	1.22 × 10^−07^	1.52 × 10^−03^
Vitamin B12	− 1.28 × 10^−04^	−2.14 × 10^−02^	1.64 × 10^−02^	

Rows are sorted by the absolute value of elastic net coefficients

We identified magnesium, calcium, high density lipoprotein (HDL-C), phosphorus, chloride, free triiodothyronine, albumin, insulin, and uric acid significant in diabetic subjects using the elastic net and glinternet. We identified magnesium, high density lipoprotein (HDL-C), chloride, free triiodothyronine, insulin, and uric acid (P values $3.35\times 10^{-10}$, $3.73\times 10^{-03}$, $2.99\times 10^{-09}$, $2.58\times 10^{-03}$, $1.88\times 10^{-04}$, and $1.31\times 10^{-05}$ respectively) as significant variables using the lasso proj. We identified magnesium, high density lipoprotein (HDL-C), chloride, insulin, and uric acid (P values $2.34\times 10^{-09}$, $6.96\times 10^{-04}$, $7.43\times 10^{-11}$, $9.36\times 10^{-02}$, and $4.05\times 10^{-04}$ respectively) as significant variables using hdi.

Similarly, we identified magnesium, high density lipoprotein, albumin, calcium, c-peptide of insulin, cholesterol, total bilirubin, vitamin D, triglycerides, uric acid, and vitamin B12 significant in obese subjects using the elastic net and glinternet. We identified high density lipoprotein, albumin, cholesterol, vitamin D, uric acid, and vitamin B (P values $7.46\times 10^{-03}$, $1.11\times 10^{-05}$, $1.03\times 10^{-03}$, $1.22\times 10^{-07}$, and $1.64\times 10^{-02}$ respectively) as significant variables using the lasso proj. We identified albumin and uric acid (P values $2.40\times 10^{-09}$ and $1.52\times 10^{-03}$ respectively) as significant variables using hdi.

### Machine learning models

**Fig. 2:**
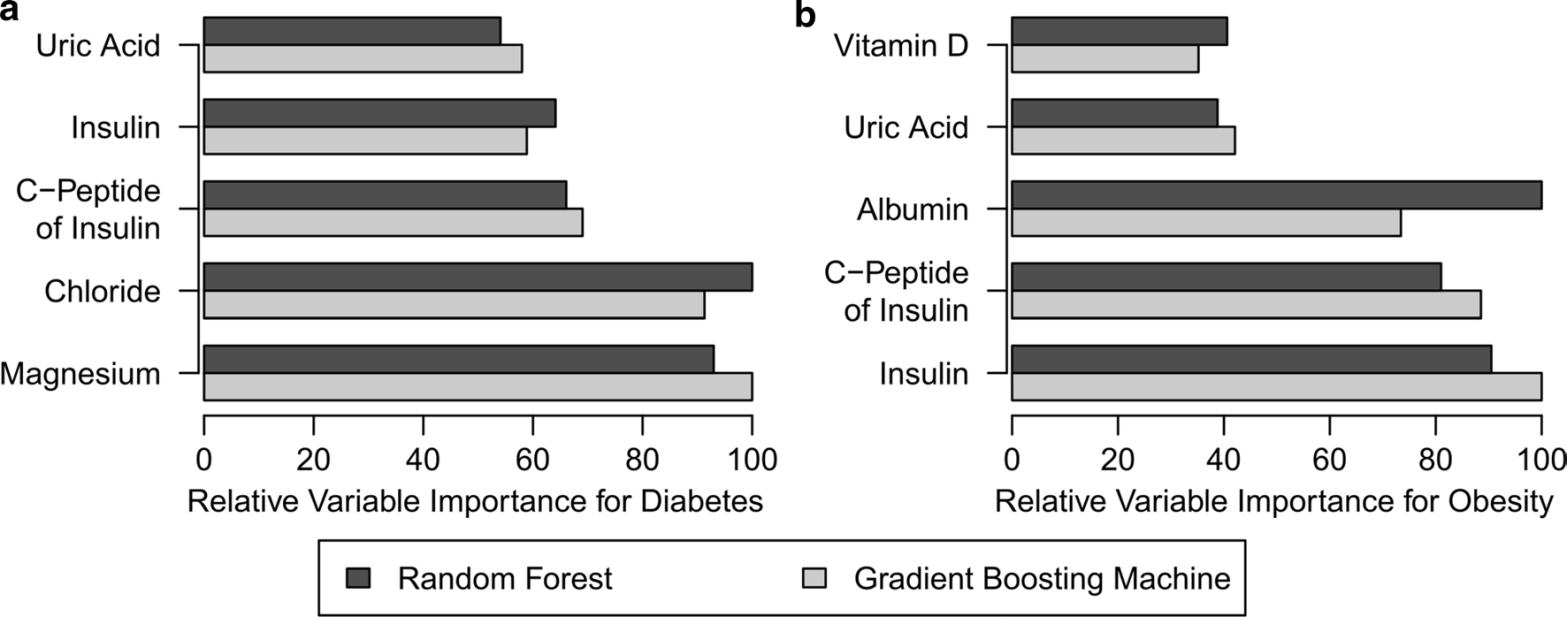
Relative variable importance of top variables of machine learning methods for (**a**) diabetes and (**b**) obesity

Results of machine learning models are summarized in Fig. 2. For diabetes study, both random forest and GBM have identified magnesium, chloride, c-peptide of insulin, insulin, and uric acid as important variables for predicting diabetes. Similarly, insulin, c-peptide of insulin, albumin, uric acid, and vitamin D were identified as main variables for predicting obesity.

**Fig. 3:**
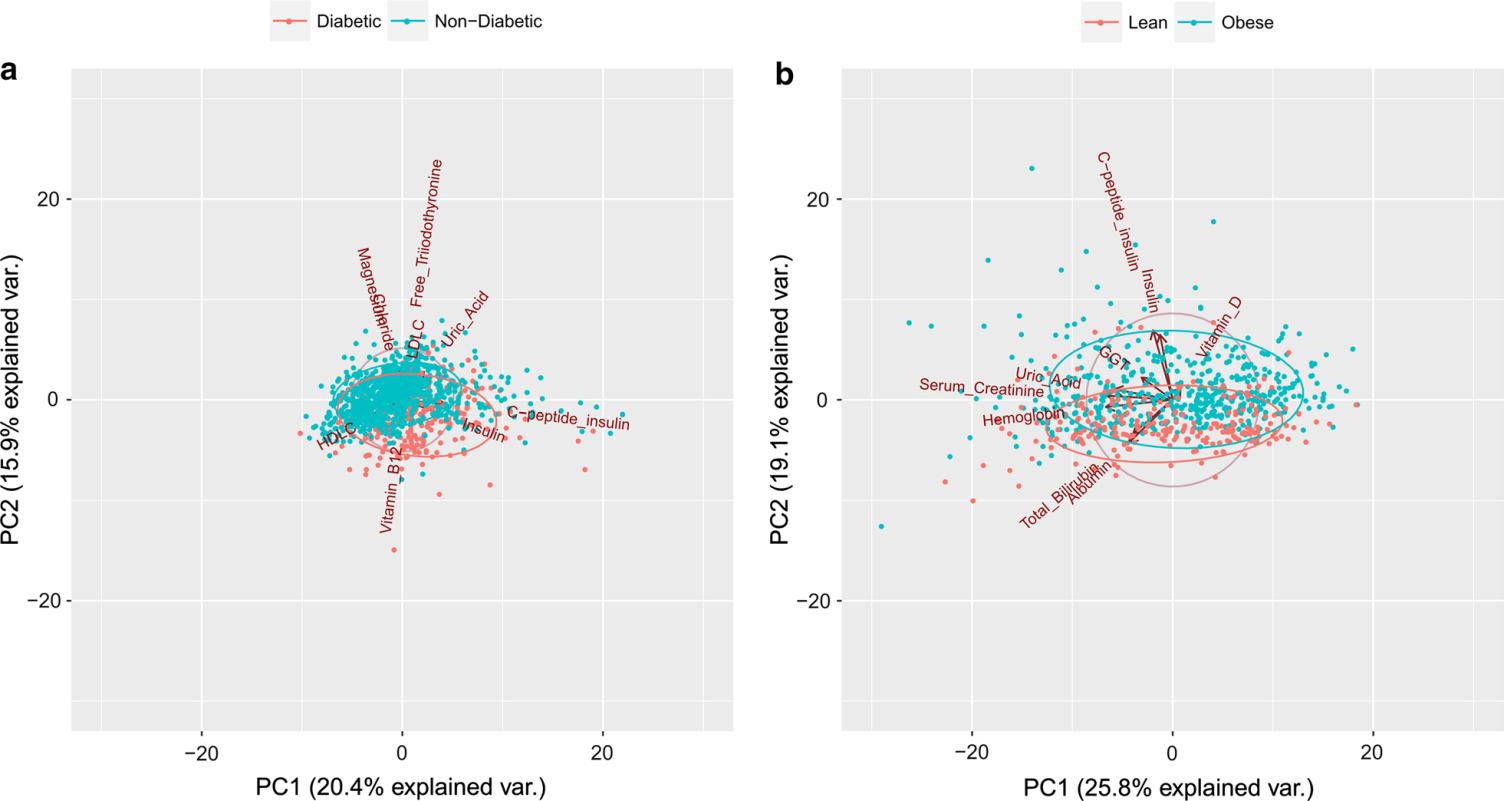
PCA Biplots for (**a**) diabetes and (**b**) obesity studies

The PCA biplots of first two principal components (PCs) are shown in Fig. 3. The plots indicate that there are overlapping clusters of cases and controls detected by the first two principal components, which is expected especially in case of diabetes indicating presence of pre-diabetic subjects. For diabetes study, there is a high correlation between magnesium and chloride; free triiodothyronine and LDLC; and c-peptide of insulin and insulin (Fig. 3a). Similarly, for the obesity study there is a high correlation between c-peptide of insulin and insulin; total bilirubin and albumin; and hemoglobin, serum creatinine and uric acid (Fig. 3b).

### Survival and risk analysis

#### Survival analysis

**Fig. 4:**
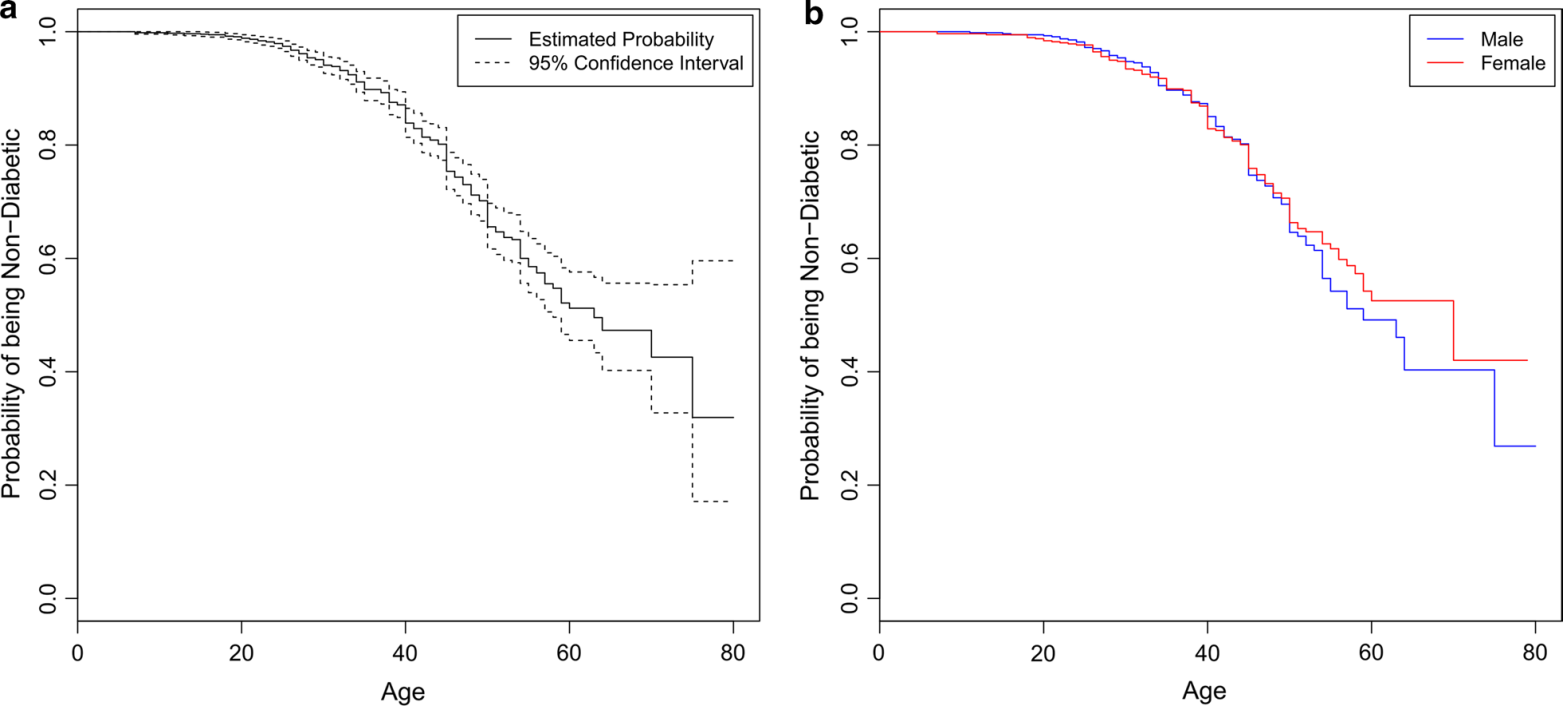
Probability of being non-diabetic (y-axis) in Qatari population (**a**) at a given age (x-axis), and (**b**) stratified on gender

Figure 4a shows the probability of being non-diabetic (y-axis) in Qatari population at a given age (x-axis). In the plot, the solid line indicates the probability of being non-diabetic (solid line) along with the $95\%$ confidence intervals (dotted lines). Variation in the probability increases with age due to a large number of uncensored observations thus widening the $95\%$ confidence interval associated with the probability. The analysis reveals that at the age of 40, there are $15\%$ chances of developing diabetes in Qatari population and the chances increase to $50\%$ at the age of 63. We have also analyzed the data by stratifying on the basis of gender. Figure 4b shows the probability of being non-diabetic (y-axis) in Qatari population at a given age (x-axis) for males and females. The results indicate that females are slightly at more risk to diabetes than males before the age of 40 but later on males have more chances to develop diabetes.

#### Risk analysis

**Fig. 5:**
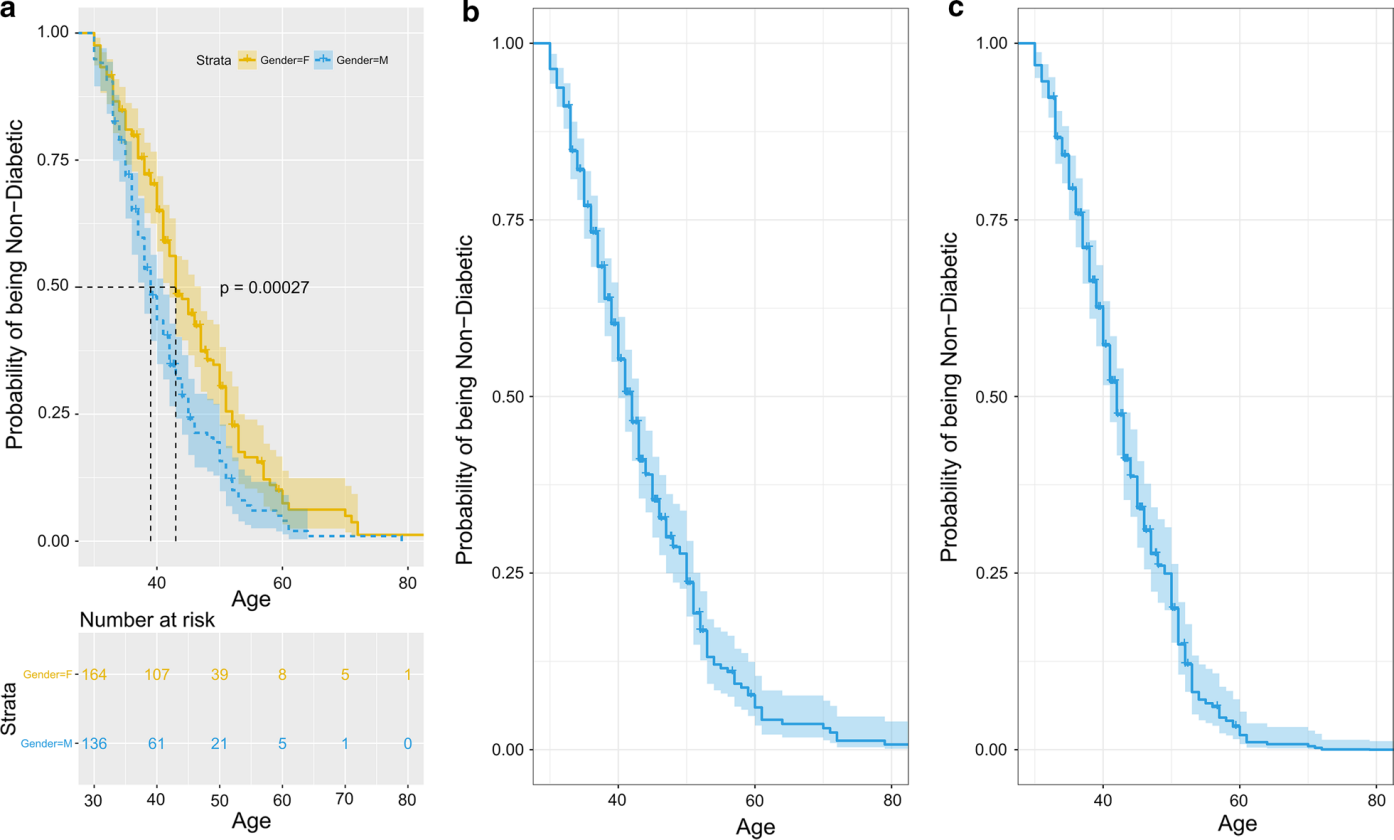
Impact on survival probability of (**a**) gender, (**b**) magnesium, and (**c**) all variables

We have performed cox proportional hazard regression analysis for each of the predictor variable independent of the other. The results are summarized in Table 3. Here lower p-values, high magnitude of $\beta$, and high value of Wald test means a variable is playing an important role in the risk of disease. In this case, variables such as calcium, magnesium, hemoglobin, triglycerides, and free-triiodothrymine play a very significant role in determining risk of the disease. The proportionality assumption of each variable must be validated in the model for correct modeling of the data. We have used scaled Schoenfeld Residuals test [31] to check proportionality assumption of each variable. Results of the test are summarized in Additional file 4. Only triglycirides variable violates the proportionality assumption as its p-value is less than the 0.05 threshold. We have investigated the impact of gender and magnesium on the survival as shown in Fig. 5a, b. We have also performed the multivariate cox regression on all the variables together in a multivariate regression setting. The results are shown in Fig. 5c.

**Table 3:** Multivariate Cox regression results for diabetes

Variable	*β*	HR (95% CI for HR)	Wald test	P value
Hemoglobin	1.7 × 10^−1^	1.2 (1.1–1.3)	20.0	9.0 × 10^−6^
Albumin	9.9 × 10^−2^	1.1 (1.1–1.2)	18.0	1.9 × 10^−5^
ALT (GPT)	1.5 × 10^−02^	1.0 (1.0–1.0)	15.0	8.7 × 10^−5^
HDLC	− 7.2 × 10^−1^	0.48 (0.33–0.71)	14.0	2.1 × 10^−4^
Gender	− 4.5 × 10^−1^	0.64 (0.5–0.81)	13.0	3.5 × 10^−4^
Total bilirubin	5.8 × 10^−02^	1.1 (1.0–1.1)	8.7	3.2 × 10^−3^
GGT	4.0 × 10^−03^	1.0 (1.0−1.0)	7.2	7.3 × 10^−3^
Free triiodothyronine	1.9 × 10^−01^	1.2 (1.0–1.4)	6.9	8.6 × 10^−3^
AST (GOT)	1.6 × 10^−01^	1.0 (1.0−1.3)	6.2	1.3 × 10^−2^
LDLC	1.6 × 10^−01^	1.2 (1.0–1.3)	6.0	1.4 × 10^−2^
Triglycerides	1.5× 10^−01^	1.2 (1.0–1.3)	5.3	2.1 × 10^−2^
Calcium	1.4 × 10^+0^	4.1 (1.1–16.0)	4.2	4.1 × 10^−2^
ALP	− 5.9 × 10^−03^	0.99 (0.99–1.0)	3.9	4.7 × 10^−2^
Magnesium	1.5 × 10^+0^	4.3 (1.0–18.0)	3.9	4.8 × 10^−2^

Rows are sorted by the P values

## Discussion

A majority of adults in Qatar are obese or overweight, which is a main risk factor for developing diabetes and between 18.5 and 20% population have been diagnosed with diabetes, according to Qatar Diabetes Association of Qatar Foundation. Both conditions—which are related to each other as well as to heart disease-increased significantly in just 6 years, with the prevalence of diabetes alone jumping nearly $20\%$ between 2012 and 2016. Although there are a number of factors associated with diabetes and obesity, ranging from genetics to individual behaviors, the metabolomics and other factors have been increasingly implicated in these epidemics. Our study is based on a new data from the 2015 to 2016 Biobank Health Interview Survey, the nation’s largest health survey.

The study proposes use of state of the art statistical and machine learning methods to identify biomarkers for medical conditions; diabetes and obesity in this case. The statistical methods rely on lasso and group-lasso based techniques that can even use mixed continuous and categorical variables. The machine learning methods rely on tree based models that provide importance of variables in predictions. In contrast to relying solely on the widely used baseline statistics, which perform marginal analysis considering a single variable at a time, these methods are based on multivariate analysis of the medical conditions. Moreover, we recommend using an ensemble of methods complementing their findings. This is because some variables are either identified by only some methods such as calcium, phosphorus, triglycerides (as shown in Table 2), or variable significance could vary between the methods such as magnesium, chloride, insulin (as shown in Table 2 and Fig. 2). From gender stratified analysis, we found that some variables have higher significance in gender specific groups compared to the whole dataset. In diabetes study, uric acid has high significance in males and triglycerides have high significance in females. Similarly in obesity study, insulin has high significance in males and HBA1C% has high significance in females.

According to world health organization, drinking water accounts for $29{-}38\%$ of the estimated average requirement of magnesium [32]. Nriagu et al. have found association of low mineral desalinated water with cancer [33]. Their findings of low magnesium water in $99\%$ portable water supply can be one of the contributing factors in hypomagnesia shown in both cases and controls. Recently, Gommers et al. have also found hypomagnesia to be one of the causes of type 2 diabetes [34].

Although hypomagnesemia have been reported low in diabetes, to the best of our knowledge chloride is not reported low in diabetic subjects. Low levels of magnesium and chloride may be an indicator of renal impairment [35]. Moreover, our study has revealed interactions of hypomagnesemia with HDL-C, triglycerides, and free thyroxine. These findings need further investigations. In next study, we will have available genomics and proteomics data and we intend to use a more advanced integrative analysis tools to associate these two diseases with genetics and other factors.

## Conclusion

Our study strongly confirms known associations and risk factors associated with diabetes and obesity in Qatari population as previously found in other population studies. For diabetes, biomarkers in Qatari population (as identified by different methods) include magnesium, calcium, HDL-C, chloride, insulin, c-peptide of insulin which have been previously reported by [36–40] to list a few. Similarly, for obesity, significant biomarkers (as identified by different methods) include insulin, c-peptide of insulin, albumin, and uric acid which have been previously reported by [41–44].

## Additional files

**Additional file 1.** Details of machine learning methods.

**Additional file 2.** Gender stratified analysis.

**Additional file 3.** Complete baseline characteristics for diabetes and obesity study.

**Additional file 4.** Scaled Schoenfeld Residuals test results for risk analysis.
