# Correction to: Harnessing Qatar Biobank to understand type 2 diabetes and obesity in adult Qataris from the First Qatar Biobank Project

**DOI:** 10.1186/s12967-018-1648-7

**Published:** 2018-10-15

**Authors:** Ehsan Ullah, Raghvendra Mall, Reda Rawi, Naima Moustaid-Moussa, Adeel A. Butt, Halima Bensmail

**Affiliations:** 1 Qatar Computing Research Institute, Hamad Bin Khalifa University, Doha, Qatar; 2 Vaccine Research Center, National Institute of Allergy and Infectious Diseases, National Institutes of Health, Bethesda, MD 20814 USA; 3 Obesity Research Cluster (ORC), Nutrigenomics, Infammation and Obesity Research (NIOR) Laboratory, Texas Tech University, 1301 Akron Street, Lubbock, TX 79409-1270 USA; 4 Department of Medicine, Weill Cornell Medical College, Doha, Qatar; 5 Department of Healthcare Policy and Research, Weil Cornell Medical College, Doha, Qatar; 6 Department of Medicine, Clinical Epidemiology Research Unit, Hamad Medical Corporation, Doha, Qatar

## Correction to: J Transl Med (2018) 16:99 10.1186/s12967-018-1472-0

Following publication of the original article [1], the authors reported that one of the authors’ names was processed incorrectly. In this Correction the incorrect and correct author name are shown. The original publication of this article has been corrected.

Originally the author name was published as:

- Naima M. Moustaid

The correct author name is:

- Naima Moustaid-Moussa

## References

[1] Ullah E, Mall R, Rawi R, Moustaid-Moussa M, Butt AA, Bensmail H (2018). Harnessing Qatar Biobank to understand type 2 diabetes and obesity in adult Qataris from the First Qatar Biobank Project. J Transl Med.

