# Supplementary material for: Harnessing Qatar Biobank to understand type 2 diabetes and obesity in adult Qataris from the First Qatar Biobank Project
Source: J Transl Med. 2018 Apr 12;16:99. doi: 10.1186/s12967-018-1472-0 (PMC5898076; doi:10.1186/s12967-018-1472-0)
Supplement: Supplementary file 1 — Additional file 1. Details of machine learning methods. [file 12967_2018_1472_MOESM1_ESM.pdf]

Harnessing Qatar Biobank to Understand Type 2 Diabetes and Obesity in Adult Qataris from the First Qatar Biobank Project

Ehsan Ullah, Raghvendra Mall, Reda Rawi, Naima M Moustaid,
Adeel A Butt, Halima Bensmail

Details of Machine Learning Methods

Random Forests

Given a dataset $\mathcal{D} = \{X_i, y_i\}_{i=1}^{n}$ with response $y_i \in \{0, 1\}$, bagging repeatedly selects random samples with replacement from the dataset $\mathcal{D}$ and fits separate trees to these samples. Algorithm 1 provides a summary of the random forest technique.

Algorithm 1: Random Forest Algorithm

for $t = 1 : T$ **do**
  Sample, with replacement, m examples from $\mathcal{D}$ to get $\mathcal{D}_t$.
  Cross-validate and build a decision tree: $\hat{f}_t$ on $\mathcal{D}_t$.
  After cross-validating, predictions for unseen samples X' can be made by taking a majority vote from all the individual decision trees on X'.
end

The maximum number of iterations T used were 1000. The number of predictors used in the model was the only parameter, which was tuned by selecting different number of predictors through random trials and using root mean square error (RMSE) for the model as evaluation metric. RMSE for different number of predictors is plotted in Figure 1. The number of predictors corresponding to the minimum RMSE was used for training the models.

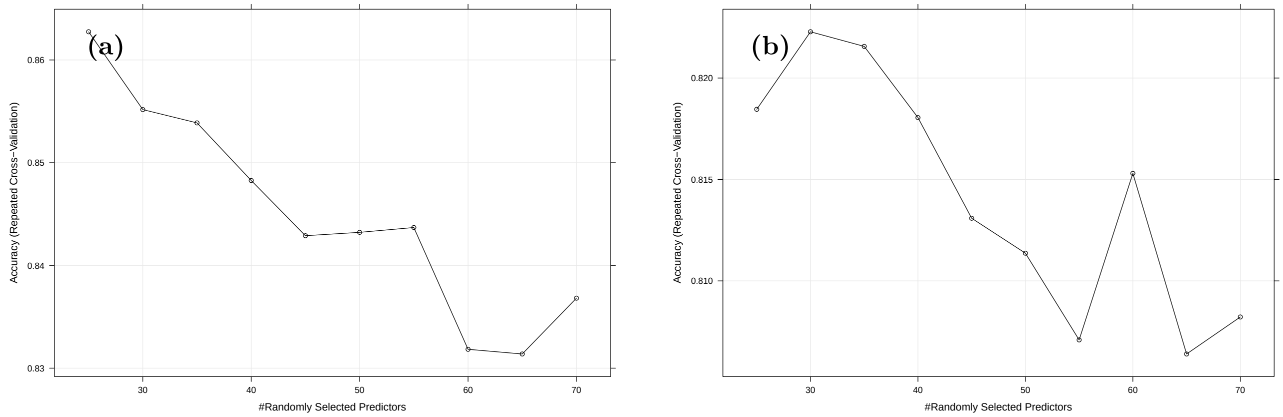

Figure 1: Random forest tuning parameters for (a) diabetes study and (b) obesity study.

Gradient Boosting Machine

This boosting method is based on a constructive strategy that the learning procedure will consecutively fit new models to provide a more accurate estimate of the response variable. The principle idea behind this algorithm is to construct the new base-learners to be maximally correlated with the negative gradient of the loss function, associated with the whole ensemble. Any arbitrary loss function ($L(\cdot,\cdot)$) can be used here. However, if the error function is the classic squared-error loss, the learning procedure would result in consecutive error-fitting. Algorithm 2 briefly summarizes the GBM technique.

Algorithm 2: Gradient Boosting Machine

Input: $\mathcal{D} = \{X_i, y_i\}_{i=1}^{n}$, a differentiable loss function $L(y, F(X))$ and number of iterations T.

Initialize model: $F_0(X) = \text{argmin}_\gamma \sum_{i=1}^{n} L(y_i, \gamma)$.

for $t = 1$ *to* T **do**

  Compute the *pseudo-residuals*: $r_{it} = -\left[\frac{\partial L(y_i, F(X_i))}{\partial F(X_i)}\right]_{F(X)=F_{t-1}(X)}, \forall t = 1, \ldots, n.$

  Fit a new base learner $h_t(X)$ on the revised dataset $\{X_i, r_{it}\}_{i=1}^{n}$.

  Compute the parameter γ_t by solving the line-search problem:

$$\gamma_t = \text{argmin}_\gamma \sum_{i=1}^{n} L(y_i, F_{t-1}(X) + \gamma_t h_t(X)).$$

  Update the model: $F_t(X) = F_{t-1}(X) + \gamma_t h_t(X)$.

end

Output: $F_t(X)$

The GBM has two tunable parameters: maximum tree depth and the number of boosting iterations. We used different values of maximum tree depth and tried different number of boosting iterations using RMSE as evaluation metric of the models through repeated cross-validation. RMSE for different number of predictors is plotted in Figure 2. Although RMSE tend to plateau with increasing number of boosting iterations, we used the maximum tree depth and the number of iterations corresponding to which the minimum is achieved.

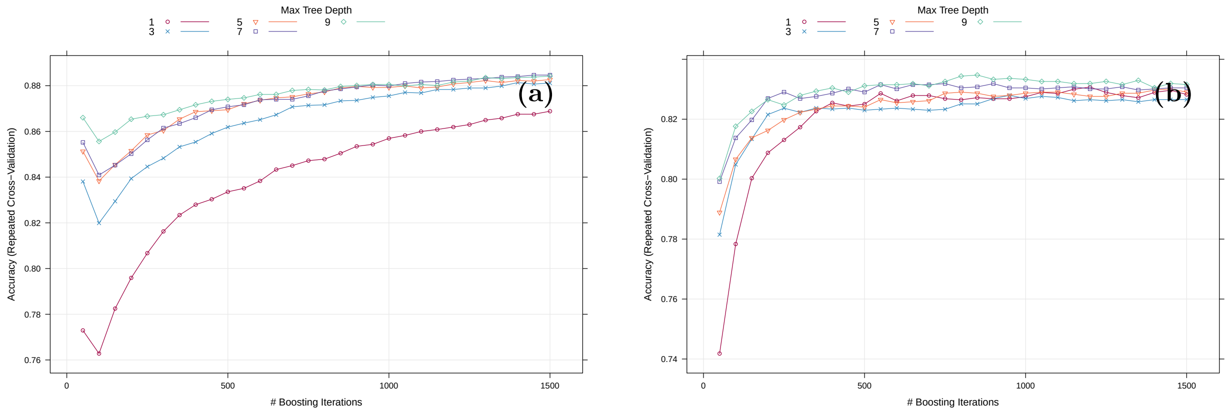

Figure 2: Gradient boosting machine tuning parameters for (a) diabetes study and (b) obesity study.
