# Supplementary material for: Harnessing Qatar Biobank to understand type 2 diabetes and obesity in adult Qataris from the First Qatar Biobank Project
Source: J Transl Med. 2018 Apr 12;16:99. doi: 10.1186/s12967-018-1472-0 (PMC5898076; doi:10.1186/s12967-018-1472-0)
Supplement: Supplementary file 2 — Additional file 2. Gender stratified analysis. [file 12967_2018_1472_MOESM2_ESM.pdf]

Harnessing Qatar Biobank to Understand Type 2 Diabetes and Obesity in Adult Qataris from the First Qatar Biobank Project

Ehsan Ullah, Raghvendra Mall, Reda Rawi, Naima M Moustaid,
Adeel A Butt, Halima Bensmail

Gender Stratified Analysis

Baseline Statistics

Table 1: Baseline characteristics for males in diabetes study. (Rows are sorted by significance)

	Diabetic ($n = 160$)	Non-Diabetic ($n = 454$)	P-value
Age	48.92 ± 10.69	38.51 ± 11.51	5.54×10^{-22}
Magnesium	0.8 ± 0.07	0.85 ± 0.06	4.92×10^{-18}
Chloride	99.17 ± 2.5	101.02 ± 1.93	2.72×10^{-15}
Uric Acid	298.29 ± 78.49	351.46 ± 67.79	5.25×10^{-13}
Sodium	139.28 ± 2.37	140.74 ± 2.13	1.33×10^{-12}
Albumin	45.12 ± 2.6	46.46 ± 2.56	2.24×10^{-08}
Free triiodothyronine	4.4 ± 0.58	4.68 ± 0.59	1.81×10^{-07}
Folate Serum	25.84 ± 7.5	23.3 ± 7.81	3.79×10^{-04}
High Density Lipoprotein (HDL-C)	1.1 ± 0.29	1.19 ± 0.29	5.70×10^{-04}
Triglycerides	1.93 ± 1.09	1.58 ± 1.19	9.18×10^{-04}
Total Bilirubin	7.18 ± 3.41	8.31 ± 5.06	1.74×10^{-03}
Homocysteine	9.04 ± 2.66	9.85 ± 3.42	2.27×10^{-03}
Insulin	26.11 ± 32.82	17.36 ± 24.6	2.33×10^{-03}
Serum Creatinine	74.53 ± 13.72	79.87 ± 33.35	5.17×10^{-03}
Vit. D	19.9 ± 9.75	17.61 ± 9.02	7.13×10^{-03}
BMI	29.7 ± 5.21	28.5 ± 5.09	1.11×10^{-02}
C-Reactive Protein	7.68 ± 6.18	6.33 ± 5.06	1.34×10^{-02}
Low Density Lipoprotein (LDL-C)	2.92 ± 0.96	3.11 ± 0.91	2.33×10^{-02}
GGT	42.12 ± 38.7	33.9 ± 42.62	3.20×10^{-02}
AST (GOT)	20.68 ± 8.42	22.41 ± 13.93	6.38×10^{-02}
Cholesterol	4.85 ± 1.09	5.01 ± 0.98	8.48×10^{-02}
C-Peptide of insulin	3.43 ± 2.48	3.07 ± 2.04	1.00×10^{-01}
Creatine Kinase	133.5 ± 115.99	156.66 ± 229.71	1.02×10^{-01}
Phosphorus	1.12 ± 0.18	1.13 ± 0.19	3.08×10^{-01}
Hemoglobin	14.7 ± 1.23	14.81 ± 1.07	3.33×10^{-01}
Thyroid Stimulating Hormone (TSH)	1.63 ± 1.19	1.87 ± 4.91	3.47×10^{-01}
Vitamin B12	334.45 ± 179.1	322.09 ± 161.78	4.20×10^{-01}
Alkaline Phosphatase (ALP)	71.99 ± 20.14	70.63 ± 18.74	4.41×10^{-01}
Free Thyroxine	13.21 ± 1.38	13.31 ± 1.66	4.54×10^{-01}
Calcium	2.4 ± 0.09	2.4 ± 0.09	5.69×10^{-01}
ALT (GPT)	30.11 ± 16.56	29.73 ± 22.39	8.22×10^{-01}
Potasium	4.39 ± 0.3	4.39 ± 0.32	9.49×10^{-01}

Table 2: Baseline characteristics for females in diabetes study. (Rows are sorted by significance)

	Diabetic ($n = 152$)	Non-Diabetic ($n = 444$)	P-value
Age	53.16 ± 8.8	39.53 ± 12.72	1.60×10^{-38}
Triglycerides	1.71 ± 0.81	1.19 ± 0.71	1.25×10^{-11}
Alkaline Phosphatase (ALP)	81.35 ± 21.03	68.23 ± 20.68	4.16×10^{-11}
Magnesium	0.78 ± 0.09	0.83 ± 0.06	1.07×10^{-10}
Chloride	99.72 ± 2.7	101.34 ± 2.04	1.13×10^{-10}
BMI	33.23 ± 6.13	29.61 ± 6.66	6.25×10^{-09}
Vit. D	23.95 ± 9.32	18.71 ± 9.73	1.17×10^{-08}
High Density Lipoprotein (HDL-C)	1.33 ± 0.33	1.49 ± 0.35	1.90×10^{-06}
GGT	33.32 ± 32.71	20.51 ± 20.51	1.01×10^{-05}
Albumin	43.33 ± 2.82	44.46 ± 2.81	2.08×10^{-05}
Vitamin B12	369.02 ± 247.06	285.97 ± 149.7	1.30×10^{-04}
Calcium	2.4 ± 0.1	2.37 ± 0.1	3.50×10^{-04}
Free triiodothyronine	4.2 ± 0.82	4.46 ± 0.65	3.60×10^{-04}
C-Peptide of insulin	3.17 ± 1.78	2.6 ± 1.46	4.07×10^{-04}
Insulin	27.91 ± 56.3	12.2 ± 10.28	8.00×10^{-04}
ALT (GPT)	22.19 ± 12.94	17.63 ± 17.9	8.10×10^{-04}
C-Reactive Protein	8.7 ± 5.83	7.16 ± 5.13	3.93×10^{-03}
Free Thyroxine	14.14 ± 2.44	13.72 ± 2.01	5.76×10^{-02}
Uric Acid	268.82 ± 75.94	256.39 ± 64.1	7.16×10^{-02}
Total Bilirubin	5.63 ± 2.45	6.05 ± 3.15	8.77×10^{-02}
Sodium	139.48 ± 2.71	139.85 ± 2.29	1.34×10^{-01}
Phosphorus	1.19 ± 0.15	1.17 ± 0.15	1.39×10^{-01}
Homocysteine	7.86 ± 2.59	8.2 ± 2.46	1.41×10^{-01}
AST (GOT)	18.89 ± 9.42	17.53 ± 10.32	1.50×10^{-01}
Thyroid Stimulating Hormone (TSH)	1.83 ± 1.36	2.21 ± 5.23	1.57×10^{-01}
Creatine Kinase	82.32 ± 78.87	73.44 ± 41.57	1.86×10^{-01}
Folate Serum	27.31 ± 8.07	26.37 ± 7.79	2.06×10^{-01}
Hemoglobin	12.51 ± 1.25	12.38 ± 1.25	2.77×10^{-01}
Cholesterol	5 ± 1.11	4.92 ± 0.88	4.55×10^{-01}
Potasium	4.39 ± 0.31	4.37 ± 0.37	5.09×10^{-01}
Low Density Lipoprotein (LDL-C)	2.87 ± 0.96	2.91 ± 0.79	6.54×10^{-01}
Serum Creatinine	56.97 ± 14.09	56.54 ± 8.91	7.25×10^{-01}

Table 3: Baseline characteristics for males in obesity study. (Rows are sorted by significance)

	Obese ($n = 203$)	Normal ($n = 122$)	P-value
C-Peptide of insulin	3.83 ± 2.49	2.4 ± 1.64	1.50×10^{-09}
High Density Lipoprotein (HDL-C)	1.11 ± 0.27	1.29 ± 0.29	8.65×10^{-09}
Triglycerides	1.88 ± 1.46	1.26 ± 0.67	3.29×10^{-07}
Insulin	26.35 ± 35.89	12.26 ± 13.11	7.58×10^{-07}
Albumin	45.43 ± 2.54	46.88 ± 2.67	1.78×10^{-06}
Uric Acid	347.47 ± 77.57	311.66 ± 55.68	8.47×10^{-06}
ALT (GPT)	32.43 ± 24.96	24.99 ± 14.4	7.38×10^{-04}
Total Bilirubin	7.31 ± 4.78	9.31 ± 5.64	1.25×10^{-03}
Free Thyroxine	13.06 ± 1.39	13.61 ± 1.47	1.31×10^{-03}
Age	43.12 ± 11.37	38.84 ± 13.68	4.04×10^{-03}
GGT	41.95 ± 55.7	28.55 ± 27.75	4.21×10^{-03}
HBA1C %	6.44 ± 1.73	5.94 ± 1.43	4.83×10^{-03}
Cholesterol	5.03 ± 1.02	4.72 ± 0.93	6.71×10^{-03}
Vit. D	16.77 ± 7.27	19.17 ± 10.5	2.70×10^{-02}
AST (GOT)	23 ± 14.64	20.44 ± 6.22	3.00×10^{-02}
Calcium	2.39 ± 0.09	2.42 ± 0.09	3.04×10^{-02}
Vitamin B12	306.36 ± 120.77	350.68 ± 206.4	3.22×10^{-02}
Free triiodothyronine	4.54 ± 0.49	4.67 ± 0.65	4.28×10^{-02}
Magnesium	0.83 ± 0.07	0.85 ± 0.07	4.50×10^{-02}
Low Density Lipoprotein (LDL-C)	3.08 ± 0.89	2.88 ± 0.84	4.53×10^{-02}
Glucose	6.94 ± 3.39	6.24 ± 2.94	5.91×10^{-02}
Chloride	100.56 ± 2.31	100.25 ± 2.21	2.41×10^{-01}
Potasium	4.38 ± 0.29	4.34 ± 0.34	2.68×10^{-01}
Sodium	140.18 ± 2.1	140.43 ± 2.45	3.16×10^{-01}
C-Reactive Protein	7.22 ± 6.09	6.57 ± 7.1	3.77×10^{-01}
Phosphorus	1.11 ± 0.17	1.13 ± 0.19	6.25×10^{-01}
Thyroid Stimulating Hormone (TSH)	1.63 ± 1.26	1.7 ± 2.66	7.57×10^{-01}
Serum Creatinine	77.58 ± 14.56	77.19 ± 10.62	7.79×10^{-01}
Alkaline Phosphatase (ALP)	71.64 ± 20.61	72.16 ± 19.41	8.21×10^{-01}
Homocysteine	9.76 ± 3.21	9.8 ± 4.04	9.09×10^{-01}
Creatine Kinase	150.7 ± 134.29	149.91 ± 192.61	9.67×10^{-01}
Folate Serum	23.63 ± 7.29	23.61 ± 7.77	9.83×10^{-01}
Hemoglobin	14.75 ± 1.1	14.76 ± 1.08	9.83×10^{-01}

Table 4: Baseline characteristics for females in obesity study. (Rows are sorted by significance)

	Obese (n = 305)	Normal (n = 102)	P-value
Age	46.85 ± 11.81	30.46 ± 9.6	4.28×10^{-32}
Albumin	43.16 ± 2.52	46.24 ± 2.51	1.39×10^{-23}
C-Peptide of insulin	3.15 ± 1.65	1.88 ± 0.96	8.64×10^{-19}
HBA1C %	6.58 ± 1.61	5.41 ± 0.96	7.66×10^{-17}
Triglycerides	1.43 ± 0.71	0.9 ± 0.51	2.08×10^{-14}
Uric Acid	275.72 ± 68.94	224.59 ± 50.39	4.40×10^{-14}
Alkaline Phosphatase (ALP)	76.99 ± 21.43	60.51 ± 16.62	5.79×10^{-14}
C-Reactive Protein	8.92 ± 6.06	5.62 ± 2.82	8.75×10^{-13}
High Density Lipoprotein (HDL-C)	1.38 ± 0.32	1.63 ± 0.35	1.86×10^{-10}
Glucose	6.88 ± 3.45	5.02 ± 2.13	5.41×10^{-10}
Magnesium	0.8 ± 0.07	0.84 ± 0.06	1.30×10^{-06}
Insulin	20.38 ± 40.59	8.64 ± 7.18	2.02×10^{-06}
GGT	26.47 ± 25.31	15.49 ± 17.69	2.41×10^{-06}
Chloride	100.62 ± 2.32	101.6 ± 1.96	5.03×10^{-05}
Creatine Kinase	79.54 ± 54.82	63.9 ± 27.59	2.00×10^{-04}
Total Bilirubin	5.44 ± 2.65	6.87 ± 3.53	2.39×10^{-04}
Serum Creatinine	57.77 ± 10.82	54.71 ± 7.02	1.13×10^{-03}
Calcium	2.37 ± 0.11	2.39 ± 0.08	1.57×10^{-02}
Phosphorus	1.17 ± 0.15	1.2 ± 0.13	2.16×10^{-02}
Thyroid Stimulating Hormone (TSH)	2.15 ± 2.78	1.7 ± 1.26	2.81×10^{-02}
Vit. D	20.23 ± 8.74	17.65 ± 11.53	4.00×10^{-02}
Cholesterol	4.96 ± 0.89	4.75 ± 0.82	4.75×10^{-02}
Low Density Lipoprotein (LDL-C)	2.93 ± 0.82	2.75 ± 0.74	5.50×10^{-02}
Potasium	4.37 ± 0.34	4.45 ± 0.4	7.00×10^{-02}
Hemoglobin	12.26 ± 1.33	12.5 ± 1.11	7.04×10^{-02}
Free triiodothyronine	4.35 ± 0.66	4.49 ± 0.58	7.20×10^{-02}
Free Thyroxine	13.79 ± 2.13	13.98 ± 1.7	3.56×10^{-01}
ALT (GPT)	19.09 ± 10.87	16.7 ± 32.8	4.71×10^{-01}
Homocysteine	8.34 ± 2.63	8.14 ± 2.53	4.87×10^{-01}
Folate Serum	26.47 ± 7.96	25.65 ± 7.2	4.92×10^{-01}
Sodium	139.71 ± 2.57	139.88 ± 2.11	5.12×10^{-01}
AST (GOT)	17.57 ± 6.77	18.11 ± 19.31	7.84×10^{-01}
Vitamin B12	302.49 ± 176.64	299.54 ± 184.57	8.84×10^{-01}

Regularization Methods

Table 5: Significant results of elastic net, glinternet, lasso proj and hdi. (Rows are sorted by the absolute value of elastic net coefficients)

	**elastic net coefficient (β)**	**glinternet coefficient (β)**	**lasso proj P-value**	**hdi P-value**
**Diabetes Study (Males)**				
Magnesium	-9.23×10^{-1}	$-1.50 \times 10^{+1}$	8.10×10^{-5}	1.08×10^{-3}
High Density Lipoprotein	-8.36×10^{-2}	-8.17×10^{-2}	5.78×10^{-1}	
Free triiodothyronine	-6.37×10^{-2}	-1.19×10^{-1}	6.55×10^{-4}	1.67×10^{-1}
Chloride	-4.70×10^{-2}	-1.70×10^{-1}	9.99×10^{-13}	1.96×10^{-11}
Albumin	-2.08×10^{-2}	-2.12×10^{-2}	2.36×10^{-6}	7.54×10^{-4}
Folate Serum	4.50×10^{-3}	1.07×10^{-2}	8.67×10^{-4}	1.10×10^{-1}
Uric Acid	-1.16×10^{-3}	-8.13×10^{-3}	5.10×10^{-14}	1.17×10^{-7}
Insulin	4.94×10^{-4}	6.76×10^{-6}	3.94×10^{-1}	
**Diabetes Study (Females)**				
Magnesium	-8.66×10^{-1}	$-2.91 \times 10^{+0}$	1.44×10^{-4}	2.09×10^{-4}
Calcium	8.47×10^{-2}	5.67×10^{-1}	2.72×10^{-1}	
Triglycerides	6.83×10^{-2}	2.20×10^{-1}	4.46×10^{-2}	7.47×10^{-4}
Free triiodothyronine	-2.29×10^{-2}	$-1.05 \times 10^{+0}$	9.26×10^{-4}	
Chloride	-1.82×10^{-2}	1.93×10^{-2}	6.98×10^{-4}	2.05×10^{-1}
High Density Lipoprotein	-1.36×10^{-2}	-4.62×10^{-1}	6.17×10^{-1}	
BMI	2.92×10^{-3}	-1.47×10^{-3}	7.12×10^{-1}	
Vitamin D	2.89×10^{-3}	3.62×10^{-3}	1.33×10^{-1}	9.41×10^{-1}
**Obesity Study (Males)**				
High Density Lipoprotein	-2.18×10^{-1}	4.05×10^{-1}	7.94×10^{-4}	1.09×10^{-1}
C-Peptide of insulin	2.31×10^{-2}	3.60×10^{-1}	6.92×10^{-2}	2.99×10^{-1}
Albumin	-1.69×10^{-2}	4.52×10^{-3}	4.38×10^{-1}	6.30×10^{-1}
Free Thyroxine	-1.61×10^{-2}	-1.40×10^{-1}	4.50×10^{-1}	
HBA1C %	1.22×10^{-2}	9.83×10^{-2}	2.06×10^{-1}	
Cholesterol	5.70×10^{-3}	-7.58×10^{-2}	3.71×10^{-1}	
Triglycerides	2.62×10^{-3}	4.12×10^{-3}		
Vitamin D	-1.22×10^{-3}	-5.27×10^{-2}	5.18×10^{-1}	
Uric Acid	8.29×10^{-4}	1.21×10^{-3}	3.23×10^{-5}	7.39×10^{-2}
ALT (GPT)	7.78×10^{-4}	-2.11×10^{-2}		
Total Bilirubin	-2.65×10^{-4}	3.93×10^{-3}		
**Obesity Study (Females)**				
High Density Lipoprotein	-5.24×10^{-2}	1.90×10^{-2}	1.84×10^{-1}	
Albumin	-4.12×10^{-2}	-1.44×10^{-1}	1.90×10^{-9}	3.87×10^{-10}
Triglycerides	3.78×10^{-2}	-3.07×10^{-1}		8.12×10^{-1}
C-Peptide of insulin	2.03×10^{-2}	-2.32×10^{-1}	2.57×10^{-1}	
HBA1C %	1.93×10^{-2}	-3.57×10^{-1}	1.55×10^{-2}	
Potassium	-1.46×10^{-2}	-2.41×10^{-2}		
Alkaline Phosphatase	1.84×10^{-3}	4.31×10^{-3}	5.65×10^{-2}	3.86×10^{-1}
Chloride	-1.49×10^{-3}	-6.24×10^{-3}	1.03×10^{-1}	
Phosphorus	-1.27×10^{-3}	-1.02×10^{-1}	3.62×10^{-1}	
Uric Acid	8.94×10^{-4}	2.14×10^{-3}	3.39×10^{-5}	6.79×10^{-4}

Machine Learning Methods

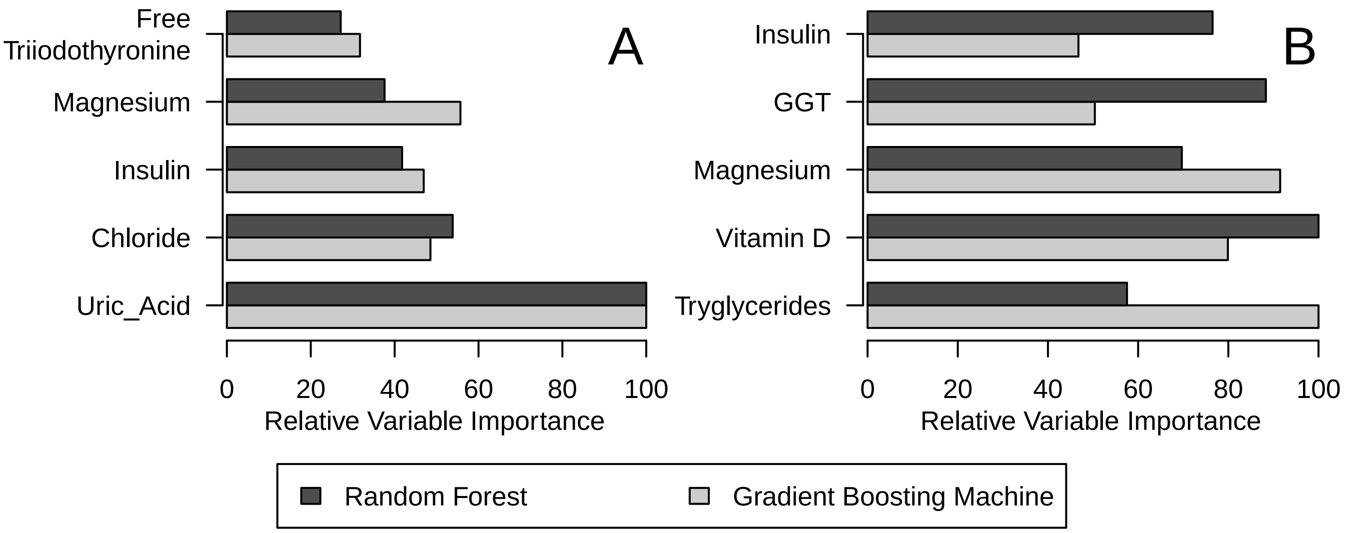

Figure 1: Relative variable importance of top variables of machine learning methods for (A) Males and (B) Females in Diabetes studies.

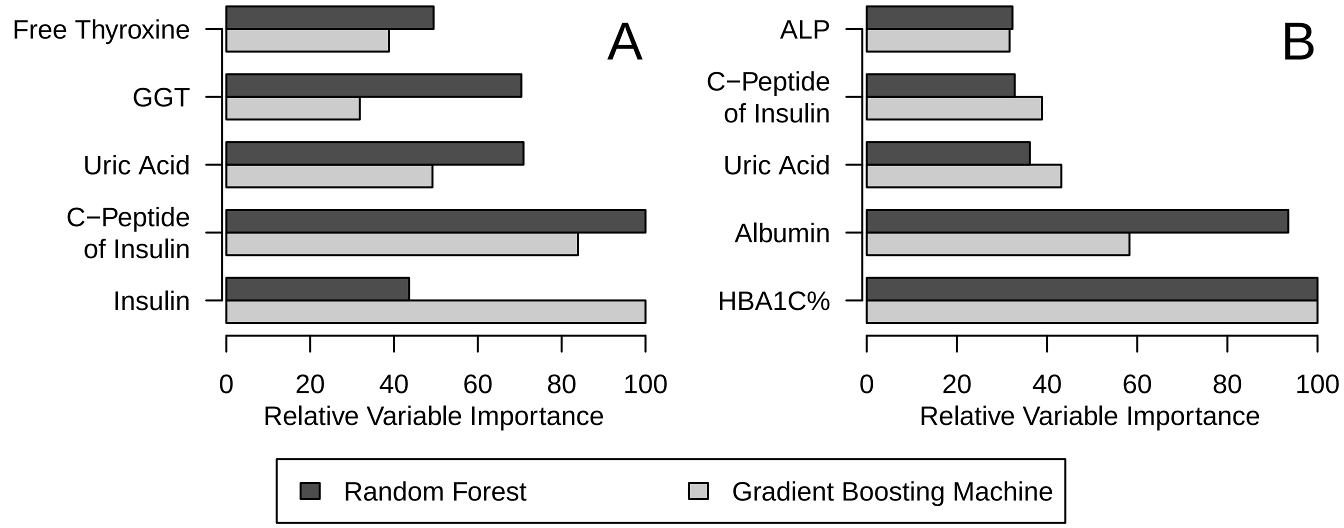

Figure 2: Relative variable importance of top variables of machine learning methods for (A) Males and (B) Females in Obesity studies.
