# Supplementary material for: Harnessing Qatar Biobank to understand type 2 diabetes and obesity in adult Qataris from the First Qatar Biobank Project
Source: J Transl Med. 2018 Apr 12;16:99. doi: 10.1186/s12967-018-1472-0 (PMC5898076; doi:10.1186/s12967-018-1472-0)
Supplement: Supplementary file 3 — Additional file 3. Complete baseline characteristics for diabetes and obesity study. [file 12967_2018_1472_MOESM3_ESM.pdf]

Harnessing Qatar Biobank to Understand Type 2 Diabetes and Obesity in Adult Qataris from the First Qatar Biobank Project

Ehsan Ullah, Raghvendra Mall, Reda Rawi, Naima M Moustaid, Adeel A Butt, Halima Bensmail

Baseline Statistics

Table 1: Baseline characteristics for diabetes study. (Rows are sorted by significance)

Age	50.99 ± 10.03	39.01 ± 12.13	**8.60×10^{-55}**
Chloride	99.44 ± 2.61	101.18 ± 1.99	**4.51×10^{-24}**
Magnesium	0.79 ± 0.08	0.84 ± 0.06	**3.50×10^{-23}**
Triglycerides	1.83 ± 0.96	1.39 ± 1.00	**2.03×10^{-11}**
Albumin	44.25 ± 2.85	45.47 ± 2.86	**1.07×10^{-10}**
BMI	31.39 ± 5.87	29.11 ± 6.00	**8.00×10^{-09}**
Free Triiodothyronine	4.31 ± 0.69	4.57 ± 0.62	**1.50×10^{-08}**
Vitamin D	21.69 ± 9.65	18.17 ± 9.40	**1.93×10^{-08}**
Sodium	139.38 ± 2.54	140.30 ± 2.25	**2.17×10^{-08}**
High Density Lipoprotein (HDL-C)	1.21 ± 0.33	1.34 ± 0.36	**5.25×10^{-08}**
Alkaline Phosphatase (ALP)	76.55 ± 21.07	69.42 ± 19.74	**8.22×10^{-08}**
Insulin	26.97 ± 45.70	14.82 ± 19.09	**7.17×10^{-06}**
GGT	37.04 ± 34.17	26.96 ± 34.05	**7.55×10^{-06}**
C-Reactive Protein	8.61 ± 7.45	6.72 ± 5.00	**3.97×10^{-05}**
Uric Acid	283.93 ± 78.53	304.45 ± 81.31	**1.13×10^{-04}**
Total Bilirubin	6.40 ± 3.03	7.19 ± 4.37	**5.17×10^{-04}**
Folate Serum	26.56 ± 7.80	24.81 ± 7.94	**7.87×10^{-04}**
C-Peptide of Insulin	3.29 ± 2.14	2.83 ± 1.80	**8.36×10^{-04}**
Vitamin B12	350.38 ± 215.53	307.04 ± 162.24	**1.26×10^{-03}**
Calcium	2.40 ± 0.09	2.39 ± 0.10	**2.46×10^{-03}**
Homocysteine	8.49 ± 2.73	8.98 ± 3.05	**8.17×10^{-03}**
ALT (GPT)	26.25 ± 15.40	23.75 ± 21.17	**2.60×10^{-02}**
Low Density Lipoprotein (LDL-C)	2.89 ± 0.96	3.01 ± 0.86	5.78×10^{-02}
Serum Creatinine	65.97 ± 16.43	68.33 ± 27.15	6.92×10^{-02}
Thyroid Stimulating Hormone (TSH)	1.71 ± 1.23	2.04 ± 5.07	6.98×10^{-02}
Free Thyroxine	13.66 ± 2.02	13.51 ± 1.85	2.30×10^{-01}
Creatine Kinase	108.01 ± 111.32	118.72 ± 200.94	2.45×10^{-01}
Cholesterol	4.92 ± 1.10	4.96 ± 0.93	5.13×10^{-01}
Potasium	4.39 ± 0.31	4.38 ± 0.35	6.66×10^{-01}
AST (GOT)	19.81 ± 8.95	20.00 ± 12.51	7.73×10^{-01}
Hemoglobin	13.63 ± 1.66	13.60 ± 1.68	7.97×10^{-01}
Gender	m = 160 ± f = 152	m = 454 ± f = 444	8.25×10^{-01}
Phosphorus	1.15 ± 0.17	1.15 ± 0.17	9.52×10^{-01}

Table 2: Baseline characteristics for obesity study. (Rows are sorted by significance)

	**Obese** ($n = 508$)	**Normal** ($n = 224$)	**P-value**
Albumin	44.07 ± 2.76	46.58 ± 2.61	$\mathbf{1.95{\times}10^{-28}}$
Age	45.36 ± 11.77	35.02 ± 12.68	$\mathbf{6.94{\times}10^{-25}}$
C-Peptide of Insulin	3.43 ± 2.07	2.17 ± 1.39	$\mathbf{1.43{\times}10^{-20}}$
Triglycerides	1.61 ± 1.10	1.10 ± 0.62	$\mathbf{5.19{\times}10^{-15}}$
HBA1C%	6.53 ± 1.65	5.71 ± 1.26	$\mathbf{6.87{\times}10^{-13}}$
Insulin	22.77 ± 38.85	10.59 ± 10.95	$\mathbf{1.54{\times}10^{-10}}$
High Density Lipoprotein (HDL-C)	1.27 ± 0.33	1.45 ± 0.36	$\mathbf{3.24{\times}10^{-10}}$
Magnesium	0.81 ± 0.07	0.84 ± 0.06	$\mathbf{3.61{\times}10^{-08}}$
Uric Acid	304.39 ± 80.52	272.01 ± 68.71	$\mathbf{4.25{\times}10^{-08}}$
Total Bilirubin	6.19 ± 3.76	8.23 ± 4.94	$\mathbf{7.18{\times}10^{-08}}$
Glucose	6.90 ± 3.42	5.68 ± 2.67	$\mathbf{2.79{\times}10^{-07}}$
Alkaline Phosphatase (ALP)	74.88 ± 21.26	66.86 ± 19.06	$\mathbf{1.47{\times}10^{-06}}$
C-Reactive Protein	8.26 ± 6.12	6.27 ± 5.10	$\mathbf{6.41{\times}10^{-06}}$
GGT	32.80 ± 41.32	22.93 ± 24.53	$\mathbf{6.67{\times}10^{-05}}$
Gender	m = 203 ± f = 305	m = 122 ± f = 102	$\mathbf{2.62{\times}10^{-04}}$
Calcium	2.38 ± 0.10	2.41 ± 0.09	$\mathbf{3.07{\times}10^{-04}}$
Cholesterol	4.99 ± 0.95	4.73 ± 0.88	$\mathbf{7.41{\times}10^{-04}}$
Hemoglobin	13.25 ± 1.74	13.73 ± 1.57	$\mathbf{7.42{\times}10^{-04}}$
Free Triiodothyronine	4.44 ± 0.61	4.56 ± 0.60	$\mathbf{1.06{\times}10^{-02}}$
Low Density Lipoprotein (LDL-C)	2.99 ± 0.85	2.82 ± 0.79	$\mathbf{1.13{\times}10^{-02}}$
Free Thyroxine	13.50 ± 1.90	13.78 ± 1.59	$\mathbf{4.00{\times}10^{-02}}$
ALT (GPT)	24.42 ± 19.03	21.21 ± 24.84	$8.61{\times}10^{-02}$
Vitamin B12	305.97 ± 156.28	329.45 ± 196.98	$1.15{\times}10^{-01}$
Sodium	139.90 ± 2.40	140.18 ± 2.31	$1.37{\times}10^{-01}$
Chloride	100.60 ± 2.32	100.86 ± 2.20	$1.44{\times}10^{-01}$
Thyroid Stimulating Hormone (TSH)	1.94 ± 2.31	1.70 ± 2.13	$1.95{\times}10^{-01}$
Folate Serum	25.34 ± 7.82	24.54 ± 7.57	$1.99{\times}10^{-01}$
Phosphorus	1.15 ± 0.16	1.16 ± 0.17	$2.08{\times}10^{-01}$
Serum Creatinine	65.69 ± 15.78	66.95 ± 14.47	$3.05{\times}10^{-01}$
Homocysteine	8.87 ± 2.93	9.08 ± 3.53	$4.30{\times}10^{-01}$
Creatine Kinase	106.70 ± 90.63	113.89 ± 164.56	$5.39{\times}10^{-01}$
Potasium	4.37 ± 0.32	4.39 ± 0.37	$5.59{\times}10^{-01}$
Vitamin D	18.90 ± 8.48	18.46 ± 10.99	$5.96{\times}10^{-01}$
AST (GOT)	19.74 ± 10.95	19.38 ± 13.83	$7.29{\times}10^{-01}$
