# Supplementary material for: Harnessing Qatar Biobank to understand type 2 diabetes and obesity in adult Qataris from the First Qatar Biobank Project
Source: J Transl Med. 2018 Apr 12;16:99. doi: 10.1186/s12967-018-1472-0 (PMC5898076; doi:10.1186/s12967-018-1472-0)
Supplement: Supplementary file 4 — Additional file 4. Scaled Schoenfeld Residuals test results for risk analysis. [file 12967_2018_1472_MOESM4_ESM.pdf]

Harnessing Qatar Biobank to Understand Type 2 Diabetes and Obesity in Adult Qataris from the First Qatar Biobank Project

Ehsan Ullah, Raghvendra Mall, Reda Rawi, Naima M Moustaid,
Adeel A Butt, Halima Bensmail

Survival Analysis

Table 1: Results of scaled Schoenfeld test to validate proportionality assumption

Variable	rho	chisq	P-value
Hemoglobin	0.01	0.01	0.93
Folate Serum	-0.01	0.01	0.93
Total Bilirubin	-0.01	0.02	0.90
BMI	0.01	0.02	0.89
C Reactive Protein	0.01	0.03	0.86
Albumin	-0.02	0.08	0.77
Creatine Kinase	-0.02	0.09	0.77
LDLC	0.02	0.09	0.76
Free Thyroxine	-0.02	0.11	0.74
Potassium	-0.03	0.17	0.68
Sodium	-0.03	0.18	0.67
ALP	0.03	0.32	0.57
ALT (GPT)	0.04	0.34	0.56
Serum Creatinine	-0.04	0.40	0.53
Cholesterol	0.04	0.43	0.51
Free Triiodothyronine	0.05	0.50	0.48
Phosphorus	-0.04	0.51	0.47
Magnesium	-0.05	0.60	0.44
Chloride	-0.06	0.61	0.43
Calcium	-0.05	0.61	0.43
TSH	0.05	0.73	0.39
AST (GOT)	0.07	0.86	0.35
Gender	0.06	0.88	0.35
Homocysteine	-0.06	1.15	0.28
HDLC	-0.07	1.20	0.27
Vitamin B12	0.07	1.28	0.26
GGT	0.10	1.95	0.16
Uric Acid	-0.10	2.26	0.13
Triglycerides	0.13	4.07	0.04
